# Understanding the long-term health effects of COVID-19

**DOI:** 10.1016/j.eclinm.2020.100586

**Published:** 2020-09-30

6 months after WHO declared it a pandemic, SARS-CoV-2 is still spreading worldwide, and COVID-19 disease has had an overwhelming impact on public health and the global economy. With more than 31 million cases reported worldwide as of Sept 21st, 2020, the number of recovered patients with persisting symptoms and unexpected sequelae is increasing. Previous outbreaks of Spanish influenza, severe acute respiratory syndrome, and Ebola have shown that survivors can suffer from long-term complications. However, research is still required to determine the long-term effects of SARS-CoV-2.

Although SARS-CoV-2 primarily affects the lungs, it has been found to damage the vascular endothelium of several other organs, resulting in complaints such as brain fog, palpitations, and fatigue, among others. The extrapulmonary manifestations of COVID-19 are varied, and the heart, brain, and kidneys are particularly susceptible to damage. This vascular component of COVID-19 might help to explain why certain patients still struggle with severe symptoms months after clearing the viral infection.

Cardiovascular disease was shown to play an important role in COVID-19 pathology early in the pandemic. Myocardial inflammation after recovery from COVID-19, even in asymptomatic or mildly symptomatic patients, has been reported. Furthermore, a study by Marc Dweck and colleagues (University of Edinburgh, Edinburgh, UK), published in the *European Heart Journal—Cardiovascular Imaging* in June, 2020, revealed that 55% of 1216 patients with COVID-19 had an abnormal echocardiogram, with evidence of left and right ventricular abnormalities, and myocardial infarction. An abnormal echocardiograph was also observed in 314 of 581 children with paediatric inflammatory multisystem syndrome associated with COVID-19, as reported in a systematic review by Mubbasheer Ahmed and colleagues (Texas Children's Hospital, Houston, TX, USA), published in *EClinicalMedicine* in September, 2020. A study by Valentina Puntmann and colleagues (University Hospital Frankfurt, Germany), published in *JAMA Cardiology* in July, 2020, suggests that there is a possibility of residual left ventricular dysfunction and ongoing inflammation months after a COVID-19 diagnosis, which might progress to heart failure and other cardiovascular complications. Additionally, by damaging the endothelium, COVID-19 might result in abnormal blood clotting, with estimates suggesting that up to 30% of people with severe COVID-19 develop blood clots. It is unclear how long the prothrombotic environment persists in recovered patients, but in the July, 2020, issue of *EClinicalMedicine,* Amy Rapkiewicz and colleagues (Long Island School of Medicine, Mineola, NY, USA) showed blood clots to affect multiple organs*.*

Evidence for the various neurological presentations associated with COVID-19 is increasing. A study by Yiping Lu and colleagues (Fudan University, Fudan, China), published in the August, 2020, issue of *EClinicalMedicine*, investigated cerebral microstructural changes in recovered COVID-19 patients 3 months after infection using MRI. Neurological symptoms, such as mood change, fatigue, headache, and visual disturbance, were present in 55% of the 60 recovered patients who had COVID-19. Furthermore, significant disruption to microstructural and functional brain integrity was observed. Other studies have revealed that SARS-CoV-2 is more likely to cause thrombotic vascular events, including stroke, than other coronaviruses and the seasonal influenza. Additionally, acute neuropsychiatric symptoms have been reported in patients with COVID-19. However, because SARS-COV-2 can penetrate the brain and cause direct damage to neuronal networks, patients might experience long-term neuropsychiatric complications, which might be exacerbated by the distress caused by the infection.

Renal involvement is frequent in COVID-19, and the clinical presentation ranges from mild proteinuria to progressive acute kidney injury. SARS-CoV-2 might attack the kidneys directly, but the kidneys are also vulnerable to the uncontrolled inflammation and blood clots that are caused by the virus. The International Society of Nephrology reported that kidney abnormalities are observed in 25–50% of patients with severe COVID-19 who require hospitalisation. The number of patients who will go on to develop chronic kidney disease is currently unknown; however, a significant number might require dialysis or transplantation.

To improve our understanding of the long-term health effects of COVID-19, large-scale standardised data collection is vital. Follow-up studies of people who have been infected with SARS-CoV-2 are currently being initiated. In the UK, the Post-hospitalisation COVID-19 study, a collaboration involving 20 universities and associated National Health Service trusts, aims to follow-up 10 000 patients for 1 year to assess the impact of COVID-19 on patient recovery and health. Similar studies are also ongoing in the USA, such as the University of California, San Francisco COVID-19 (LIINC) study led by Steven Deeks, which plans to recruit 300 patients for 2 years of follow-up. Furthermore, post-mortem studies might further illuminate the complex pathological processes underlying long-term organ damage.

With millions of recovered patients potentially experiencing damage to their blood vessels, health-care systems worldwide need to develop ways of supporting people recovering from COVID-19 to avoid further straining health resources. Dedicated rehabilitation centres for people recovering from COVID-19 need to be made available to ensure people have the best recovery, resume their daily activities as quickly as possible, and decrease socioeconomic repercussions. However, the extent to which persisting symptoms will affect health systems and the labour force remains unknown.

Because the effect of SARS-CoV-2 on the vasculature is so profound, and blood vessels interact with every organ in the body, cross-disciplinary collaborations might be required to develop effective treatment strategies. Knowledge of the vascular consequences of the disease might allow physicians to intervene early, or to alter treatment strategies, and it might be possible to prevent long-term damage.

*EClinicalMedicine*

